# Physical, Nutritional, and Bioactive Properties of Mandacaru Cladode Flour (*Cereus jamacaru* DC.): An Unconventional Food Plant from the Semi-Arid Brazilian Northeast

**DOI:** 10.3390/foods11233814

**Published:** 2022-11-26

**Authors:** Ana Cristina S. Martins, Gracy Kelly V. de V. Medeiros, Jaielison Yandro P. da Silva, Vanessa B. Viera, Paternak de S. Barros, Marcos dos S. Lima, Marcelo S. da Silva, Josean F. Tavares, Yuri M. do Nascimento, Evandro F. da Silva, Juliana K. B. Soares, Evandro L. de Souza, Maria Elieidy G. de Oliveira

**Affiliations:** 1 Academic Health Unit, Center of Education and Health, Federal University of Campina Grande, Cuité 58175-000, Brazil; 2 Department of Nutrition, Health Sciences Center, Federal University of Paraíba, João Pessoa 58051-900, Brazil; 3 Department of Mechanical Engineering, Pernambuco Polytechnic School, Federal University of Pernambuco, Recife 50670-901, Brazil; 4 Department of Food Technology, Federal Institute of Sertão Pernambucano, Petrolina 56302-100, Brazil; 5 Post-Graduate Program in Bioactive Natural and Synthetic Products, Health Sciences Center, Federal University of Paraíba, João Pessoa 58051-900, Brazil; 6 Institute for Research in Drugs and Medicines—IPeFarM, Federal University of Paraíba, João Pessoa 58051-900, Brazil

**Keywords:** *Cactaceae*, drying, nutritional composition, bioactive compounds, unconventional plant food

## Abstract

In this study, we evaluated the physical, nutritional, and bioactive properties of mandacaru cladode flour (*Cereus jamacaru* DC.). The granulometric profile revealed particles with non-uniform geometry, flakiness, a rectangular tendency, and a non-homogeneous surface, with particle sizes ranging from 20 to 60 µm. The flour presented low water activity (0.423), a moisture content of 8.24 g/100 g, high ash (2.82 g/100 g), protein (5.18 g/100 g), and total carbohydrate contents (74.48 g/100 g), and low lipid contents (1.88 g/100 g). Mandacaru flour is an excellent source of insoluble dietary fiber (48.08 g/100 g), calcium (76.33%), magnesium (15.21%), and potassium (5.94%). Notably, ^1^H NMR analysis revealed the presence of N-methyltyramine. Using HPLC chromatography, glucose was identified as the predominant sugar (1.33 g/100 g), followed by four organic acids, especially malic acid (9.41 g/100 g) and citric acid (3.96 g/100 g). Eighteen phenolic compounds were detected, with relevant amounts of kaempferol (99.40 mg/100 g), myricetin (72.30 mg/100 g), and resveratrol (17.84 mg/100 g). The total phenolic compounds and flavonoids were 1285.47 mg GAE/100 g and 15.19 mg CE/100 g, respectively. The mean in vitro antioxidant activity values were higher using the FRAP method (249.45 µmol Trolox TEAC/100 g) compared to the ABTS^•+^ method (0.39 µmol Trolox TEAC/g). Finally, the ascorbic acid had a content of 35.22 mg/100 g. The results demonstrate the value of mandacaru as a little-explored species and an excellent matrix for the development of flours presenting good nutritional value and bioactive constituents with excellent antioxidant potential.

## 1. Introduction

Consumer demand for foods with a balanced nutritional composition that are capable of providing not only basic nutrition, but also additional health benefits, has stimulated the food industry to develop new sustainable and functional food products [1,2,3].

Foods classified as sustainable due to their nutritive and bioactive components can also boost the economy and improve consumer health, all while mitigating the environmental impact commonly caused by traditional monoculture [4,5]. From the perspective of agroecology, and considering the given climatic changes and the environmental challenges faced by the planet, the food industry has increased the incorporation of unconventional (alternative) ingredients in developed food products [6]. Incorporating unconventional ingredients into food products is a promising strategy to meet consumer demand for nutritious and sustainable agroecological foods [7]. In this context, the family Cactaceae presents various non-conventional food plants (NCFP) which arouse great interest in the scientific community due to their nutritional and bioactive ingredients [8,9]. 

Cactaceae, found in the Caatinga biome, is a botanical family of shrubs, trees, herbs, lianas, and subshrubs. It presents approximately 142 genera and 1400 species found throughout Canada, the USA, Mexico, and Central and South America [10,11,12]. In the Brazilian semi-arid region, cacti contribute to the sustainability of the biome as alternative forage and food sources for animals in times of drought [13]. *Cereus jamacaru* DC., or the mandacaru cactus, is resistant to drought and possesses an excellent composition of carbohydrates, soluble and insoluble fibers, vitamins, and antioxidants to provide a matrix with excellent functional properties [14]. Although it has expressive nutritional and functional potential, it is little used for human consumption. Its properties as a food matrix are little known, making commercialization and use difficult [9].

In food products, replacing wheat flour with alternative flours is of paramount importance, and cactus flour can bring interesting compositions. The substitution of wheat flour for cactus flour in the elaboration of food products can be a strategy to meet broader agroecological perspectives [6], as well as minimize climate change and the environmental challenges currently faced by the planet [15].

In general, cacti and, consequently, the flours obtained from them, have a high content of minerals, total fiber, and phenolic compounds, with a low-fat content [8,9,16]. The use of flour from different cactus species (*Pilosocereus gounellei*, *Opuntia ficus-indica* L., *Opuntia monacantha*, and *Opuntia macrorhiza* Engelm.) could improve the technological, nutritional, and bioactive aspects of new food products, such as cookies [6,17,18], cakes [19], and yogurt [20]. However, there are no studies characterizing mandacaru (*Cereus jamacaru* DC.) cladode flour, demonstrating its potential in the elaboration of other food products.

In this study, we elaborated and evaluated the physical, nutritional, and bioactive properties of mandacaru cladode flour, a potential alternative ingredient with added-value in food processing.

## 2. Materials and Methods

### 2.1. Sample Collection and Preparation of Mandacaru Cladode Flour (MF)

Mandacaru cladodes (*Cereus jamacaru* DC.) were collected in the city of Cuité, Paraíba State, Brazil (6°29′46.0″ S–36°09′34.7″ W) on three non-consecutive days in October 2020. This yielded three different batches weighing 4 kg each. The cladodes were selected considering their physical integrity (whole cladodes, without the presence of defects or diseases), and were then transported in polystyrene boxes at 5 ± 1.0 °C. Table 1 presents the physical–chemical composition of mandacaru cladode in natura. The methodologies used to determine it were the same as those described later for mandacaru cladode flour (MF). The botanical material was registered with the National System for the Management of Genetic Heritage and Associated Traditional Knowledge (SISGEN) under number A1FECD5. 

The MF was prepared in accordance with the method described by Machado et al. [6], with modifications. Initially, the cladodes were sanitized with running potable water, immersed in a sodium hypochlorite solution (0.01%, *v*/*v*) for 15 min, and cut into slices approximately 1 cm thick using a sterilized stainless-steel knife. The cladode slices were dehydrated in an oven with forced air circulation (Quimis, Q314M, Diadema, SP, Brazil) at a temperature of 55 ± 1 °C reaching up to 4% humidity, taking approximately 26 h. After drying, the mandacaru cladodes were ground in a knife mill (Willey, Solab, Piracicaba, SP, Brazil) and screened with a 230-mesh sieve on a sieve shaker (Bertel^®^, Caieiras, SP, Brazil), resulting in MF at a final yield of 5% (compared to the cladode in nature). MF was vacuum-sealed in sterile polyethylene bags (approximately 100 g per bag), wrapped in aluminum foil, and frozen (−20 ± 1 °C) until use in the analysis (for a maximum of 1 day).

### 2.2. Chemicals and Reagents

For the realized tests, the following reagents were used (with their respective degrees of purity and manufacturers): absolute ethanol (99.3%, ACS reagent), ABTS reagent (2,2′-Azino-bis(3-ethylbenzothiazoline-6-sulfonic acid) diammonium salt (≥98%, HPLC grade), aluminum chloride (95.5%, ACS reagent), ferric chloride (97%, ACS reagent), Folin–Ciocalteu (99%, ACS reagent), methanol (99.85%, ACS reagent), methanol (99.99%, HPLC grade), phosphoric acid (ACS reagent, ≥85 wt. % in H_2_O), potassium persulfate (99%, ACS reagent), sodium acetate (99.5%, ACS reagent), sodium carbonate (99.95%, ACS reagent), sodium hydroxide (95%, ACS reagent), sodium nitrite (99%, ACS reagent), sulfuric acid (95–98%, ACS reagent), TPTZ reagent (2,4,6-Tri-(2-Pyridyl)-1,3,5-Triazine Salt 1:3 with p-toluenesulfonic acid, (≥98%, HPLC grade)), Trolox (6-hydroxy-2,5,7,8-tetramethylchroman-2-carboxylic acid (97%, ACS reagent), and 2,6-Dichloroindophenol sodium salt hydrate (ACS reagent) were obtained from Sigma-Aldrich (St. Louis, MO, EUA). Ultra-purified water was used (Milli-Q^®^ Integral Water Purification System, EMD Millipore, Billerica, MA, USA). 

The following external standards (HPLC grade) were also used: sugars (glucose, fructose, maltose, and rhamnose), organic acids (acetic, butyric, citric, lactic, malic, succinic, formic, and propionic), and phenolic compounds (gallic acid, syringic acid, hesperidin, naringenin, procyanin B1, catechin, procyanidin B2, caffeic acid, chlorogenic acid, caffeic acid, coumaric acid, cyanidin 3,5-glycidine, pelargonidin 3,5-glycoside and perlagonidine 3-glycoside, epicatechin, epicatechin gallate, procyanidin A2, quercetin 3-glycoside, rutin, kaempferol 3-glycoside, petunidin 3-glycoside, trans-resveratrol and cis-resveratrol), obtained from Sigma-Aldrich (St. Louis, MO, USA).

### 2.3. MF Quality Control

To evaluate the hygienic sanitary conditions of the processed flour, the methodology recommended by the American Public Health Association [21] was used to determine the most likely number (MLN) of total coliforms (MLN/g) and thermotolerant coliforms (MLN/g). Molds, yeast, *Bacillus cereus*, *Staphylococcus* coagulase positive, and aerobic and mesophilic bacteria were expressed in colony-forming units per g (CFU/g), and the detection of *Salmonella* sp. was expressed as absent or present. 

### 2.4. Physical–Chemical Characterization of MF

The MF morphology was evaluated in accordance with the methodology described by Brito et al. [22]. For this, a nanometric layer (40–50 nm) of gold was deposited on the sample using a metallizer (Quorum SC7620, Madrid, Spain) (20–1000 μm). Images at differing magnifications (100×–4000×) were obtained via scanning electron microscopy (SEM) (TESCAN MIRA3, Brno, Czech Republic) and particle sizes were measured.

For the physicochemical characteristics: water activity at 25 °C was determined by a direct reading with Aqualab equipment (Meter^®^, AquaLab Series 4TEV, São José dos Campos, SP, Brazil); moisture content was determined by drying in a stabilized oven at 105 °C until a constant mass was obtained; ash content (fixed mineral residue—FMR) was quantified by carbonization followed by incineration in a muffle furnace (Jung^®^, model 0612, Blumenau, SC, Brazil) stabilized at 550 °C; proteins were quantified using the micro-Kjeldahl method; total carbohydrates were determined using the Fehling reduction method [23]; and fat was quantified using the Folch, Less and Sloane-Stanley method [24]. Total insoluble and soluble fiber contents were determined using an enzymatic–gravimetric method [23,25].

Macro- and microelements were quantified in MF in accordance with the methodology described by Etienne et al. [26], with modifications. An energy-dispersive spectrometer (EDS) (OXFORD, England, London) coupled to a scanning electron microscope (SEM) (TESCAN MIRA3, Brno, Czech Republic) was operated at high vacuum pressures. The operational conditions used for the EDS analysis were: zoom (1000×), working distance (15 mm), and acceleration voltage (15 Kv). The composition was explored, and the data were transferred to MS Word and Excel software and expressed in percentages.

To determine the contents of sugars (glucose, fructose, maltose, and rhamnose) and organic acids (acetic, butyric, citric, lactic, malic, succinic, formic, and propionic), MF aqueous extract was prepared. Thus, 2 g of sample was homogenized with 10 mL of ultra-purified water (Milli-Q^®^ Integral Water Purification System, EMD Millipore, Billerica, MA, USA) for 10 min in a Turratec crusher/homogenizer (TE-102-Tecnal^®^, Piracicaba, SP, Brazil). The suspension was centrifuged (4000× *g*, 15 min, at 4 °C) and the supernatant was filtered through a 0.45 μm filter (Millex Millipore, Barueri, SP, Brazil).

Subsequently, the extract was injected into a high-performance liquid chromatographic (HPLC) system, with a 1260 Infinity LC system (Agilent Technologies, Santa Clara, CA, USA) equipped with a quaternary solvent pump (model G1311C), degasser, thermostat column compartment (model G1316A), and auto-sampler (model G1329B) coupled with a diode array detector (DAD) (model G1315D), and a refractive index detector (RID) (model G1362A). During analysis, the Agilent column Hi-Plex H (300 × 7.7 mm) with a particle size of 8.0 μm and PL Hi-Plex H guard column (5 × 3 mm) (Agilent Technologies) was kept at 50 °C, the injection volume was 10 μL, the flow rate was 0.5 mL/min, the mobile phase was H_2_SO_4_ at 4.0 mM in ultrapure water, and the run time was 20 min. The data obtained were processed using OpenLAB CDS ChemStation EditionTM (Agilent Technologies). The peaks and average peak areas were used to quantify the HPLC samples, comparing their retention times with organic acid and sugar standards [27,28,29,30]. The results are expressed as g per 100 g of sample (g/100 g).

### 2.5. Acquisition of the ^1^H NMR Spectrum 

An aliquot of 20 mg MF was solubilized in 1 mL of methanol HPLC with deuterated water (9:1, *v*/*v*), the suspension was subjected to an ultrasonic bath for 30 min, was filtered, and the resulting solution (600 μL) was placed in a 5 mm diameter tube for nuclear magnetic resonance (NMR) analysis. The NMR experiments were performed on Bruker Avance Neo 500 equipment operating at 500 MHz for ^1^H NMR and at 125 MHz for ^13^C NMR (Bruker, Billerica, MA, USA). The following parameters were used to obtain the spectra sequence: lc1pngpf2; temperature: 26 °C; number of scans: 8; dummer scan: 4; recevier gain: 36; acquisition time: 1.63 s. Spectra were processed using Bruker TopSpin 4.1.1 software.

### 2.6. Antioxidant Properties of MF

The evaluation of the antioxidant properties was based on the determination of ascorbic acid, total phenolic compounds, total flavonoids, phenolic profile, and antioxidant activity in accordance with the procedures described below.

The ascorbic acid content was determined in accordance with Tillman’s method, using a 2,6-dichlorophenol-indophenol sodium dye solution. The total ascorbic acid content was expressed in mg per 100 g of the sample (mg/100 g) [23].

For the determination of total phenolics and total flavonoids, extracts were initially obtained. Thus, 2 g of MF was homogenized with 10 mL of 80% (*v/v*) methanol solution (Sigma-Aldrich, St. Louis, MA, USA) for 10 min in a mini-Turrax apparatus (Tecnal) kept at rest for 24 h, and filtered with 125 mm filter paper (Whatman^®^, GE Healthcare, Chicago, IL, USA). The determination of total phenolic content was performed in accordance with the Folin–Ciocalteu method [31]. Absorbance was measured at 765 nm with a spectrophotometer (BEL Photonics, Piracicaba, SP, Brazil). The total phenolic content was determined with a standard curve prepared with gallic acid (Sigma-Aldrich). Results are expressed as milligrams of gallic acid equivalent (GAE) per hundred grams of sample (mg GAE/100 g).

The total flavonoid content was determined in accordance with the method proposed by Zhishen et al. [32]. The absorbance of the sample was measured at 510 nm in a spectrophotometer (BEL Photonics) against a blank in the absence of extracts. The total flavonoid content was determined using a standard curve of catechin equivalents (Sigma-Aldrich) (CE). Results are expressed as catechin equivalents (CE) per 100 g of sample (mg CE/100 g).

For the phenolic profile, an extract of the sample was initially obtained from 5 g of MF homogenized in 5 mL of 80% methanol (Sigma-Aldrich), which was centrifuged for 15 min (9000× *g*, 4 °C) and filtered through a polypropylene filter with 0.45 µm membrane (Millex Millipore). Individual phenolic compounds were determined with high-performance liquid chromatography, with gradient and runtime adaptations to quantify differing phenolic classes using an Agilent 1260 Infinity System LC liquid chromatograph (Agilent Technologies, Santa Clara, CA, USA) coupled to a diode array detector (DAD)) (G1315D). The column was a Zorbax Eclipse Plus RP-C18 (100 × 4.6 mm, 3.5 μm), and the pre-column was a Zorbax C18 (12.6 × 4.6 mm, 5 μm) (Agilent). The oven temperature was 35 °C, and the injection volume was 20 μL (phase A diluted), filtered with a 0.45 μm filter (Millex Millipore). The solvent flow was 0.8 mL/min, and the new gradients used in the separation were: zero to 5 min: 5% B; 5-14 min: 23% B; 14–30 min: 50% B; 30–33 min: 80% B. Solvent A was a solution of phosphoric acid (0.1 M, pH = 2.0) and solvent B was acidified methanol with 0.5% H_3_PO_4_. Data were processed with OpenLAB CDS ChemStation Edition software (Agilent Technologies). The detection of phenolics was completed at 220, 280, 320, 360, and 520 nm. The identification and quantification were completed by comparison with external standards (Sigma-Aldrich). The results were expressed as mg of phenolic for 100 g of sample (mg/100 g) [28]. 

To determine the antioxidant activity, the extract was initially prepared: 2 g of the MF sample was homogenized with 10 mL of 80% methanol (Sigma-Aldrich) for 10 min in a mini-Turrax apparatus (Tecnal) and kept at rest for 24 h; it was then filtered through a 125 mm filter (Whatman^®^). The ability of the extracts to reduce iron was measured using the FRAP method, as described by Benzie and Strain [33] and adapted by Pulido et al. [34]. The ability of the MF extract to capture the ABTS^•+^ (2,2-azino-bis(3-ethylbenzothiazoline)-6-sulfonic acid) cation was measured using the ABTS^•+^ method, as previously described [35]. The FRAP antioxidant activity results were expressed in micromoles of Trolox equivalent antioxidant capacity (TEAC) per 100 g of sample (μmol TEAC/100 g). The ABTS^•+^ results for antioxidant activity were expressed in micromoles of Trolox equivalent antioxidant capacity (TEAC) per gram of sample (μmol TEAC/g). 

### 2.7. Statistical Analysis

All assays were performed in triplicate in two different experiments. The results are expressed as mean ± standard deviation. 

## 3. Results and Discussion

### 3.1. MF Quality Control

The MF microbiological analysis results revealed that the samples were not contaminated, indicating that the drying process provided a stable material, meeting the requirements for flours according to the Brazilian legislation [36]. The samples were thus acceptable for consumption and/or use in food processing.

### 3.2. Physicochemical and Technological Characterization of MF

**Figure 1:**
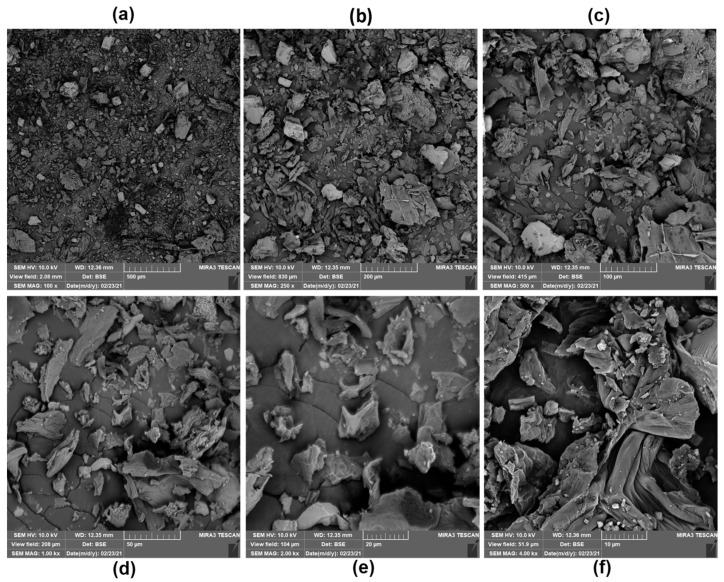
MF micrographs at 100× to 4000× magnification performed in SEM. Magnifications: (**a**) 100×, (**b**) 250×, (**c**) 500×, (**d**) 1000×, (**e**) 2000× and (**f**) 4000×.

The MF morphology (Figure 1) shows the existence of particles with a non-uniform, scaly geometry, a rectangular tendency, and a non-homogeneous surface appearance, with characteristics similar to oat flour [37]. Such aspects can be modified by the grinding process, as well as by the size of the sieve mesh, to change the quality of the flour [38,39]. In our study, we chose to use a sieve with a mesh size of 230 to produce flour with a more pulverized appearance, and to resemble more refined flours such as wheat flour. Similar results were presented by Saad et al. [40]—working with cucumber pomace flour in wheat flour-based pastas—who also used a sieve with a mesh of 230.

**Figure 2:**
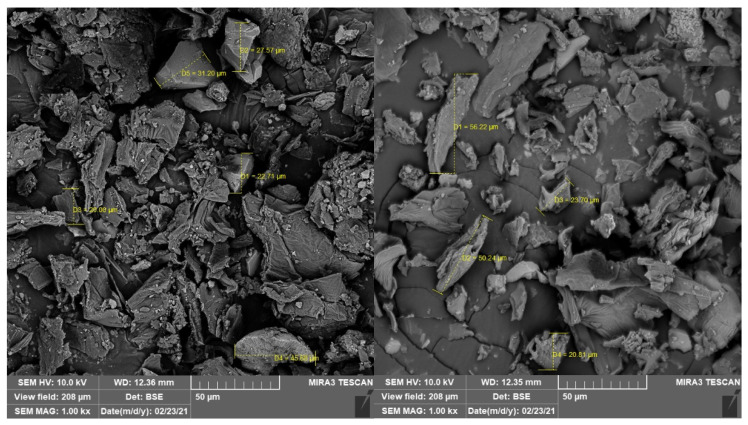
MF particle size (1000×).

The size of the flour particles depends on the raw material, processing method used (mill or processor), and mesh diameter of the sieve [41]. These affect the physicochemical composition (fibers, proteins, and starch contents), nutritional quality, sensory acceptability, and shelf life of the processed product [20,42]. In this study, a 230-mesh sieve was used, limiting the passage through the mesh of larger MF particles and providing smaller particles with diameters ranging from 20 to 56.22 µm (Figure 2). 

Due to their ability to absorb water, smaller flour particles are generally used by the food industry to replace fat and act as stabilizers and biodegradable films, as well as improve the viscosity, gelatinization, and cohesion of the dough [43,44,45]. In addition, smaller particles, such as those observed in MF, affect the thermo-mechanical properties and influence the functionality of the gluten network, yielding a smaller impact during mixing and cooking, which is a benefit for bakery products [38,42]. 

The physicochemical composition, dietary fiber content, and mineral profile of MF are shown in Table 2.

Technological processes, such as drying/dehydration, are used in the preparation of flours, generating a decrease in water activity (a_w_) and humidity, bringing benefits such as improved stability and easier transport, and minimizing greater losses in perishable foods [46]. In this study, we verified that there was an expected reduction in a_w_ and moisture in MF when compared to the cladode of mandacaru in natura (from 0.937 to 0.423 and 13.20 to 8.24 g/100 g, respectively) (Table 1 and Table 2). Values of a_w_ lower than 0.7 contribute to the inhibition of microbial multiplication [47] and make this matrix microbiologically safe, with a low risk of physicochemical changes and good stability [19,48]. This corroborates with our findings, since no microbiological contamination was detected in the MF. In a previous study by Boukid et al. [49] with *Opuntia ficus indica* f. *inermis* powder, the authors found results for a_w_ of 0.36, a value close to that detected in the present study with mandacaru flour.

The moisture content in carob, peanut, rye, corn, and cassava flours is approximately 14 g/100 g [50]. Low moisture content, such as that found in MF (8.24 g/100 g), can affect crude protein and fat content (with increased concentration), prevent microbial growth and insect infestation, and directly impact shelf life, avoiding the short-term deterioration of food products [51,52]. Moisture results close to those detected by us have been reported by Nabil et al. [51] in *Opuntia ficus-indica* cladode flour, corresponding to 9.55 g/100 g.

Ash content is an important parameter when measuring the total mineral content in a food matrix. It is also an indicator of quality in terms of nutritional labeling in the processing properties of food products [53]. According to Marshall [54], the ash content in flours (whole grains, cereals, and vegetables) varies from 0.3 to 1.4 g/100 g. MF presented a high ash content (2.82 g/100 g), even higher than that reported by Petkova et al. [55], for *Ceratonia siliqua* flour (2.25 g/100 g).

In this study, MF presented high carbohydrate (74.48 g/100 g) and protein (5.18 g/100 g) contents, with low amounts of lipids (1.88 g/100 g) (Table 2). According to Argel et al. [56], legume flours such as lentils, chickpeas, peas, and beans, due to their high concentrations of carbohydrates and proteins and low amounts of lipids, can be inserted into meat products to partially reduce fat and improve health sustainably. Dick et al. [19] analyzing *Opuntia monacantha* cladode flour found results for carbohydrates (74.84 g/100 g), proteins (5.12 g/100 g), and lipids (1.72 g/100 g) close to those found for MF.

Cactus, due to its low fat content, can be considered a sustainable alternative for the elaboration of food products [19,57]. Cactus flours have less overall lipid oxidation, making them an excellent alternative for dairy product supplements [58]. Cacti can also improve the technological, nutritional, and sensory characteristics of breads [59], cakes [20], and biscuits [6].

Dietary fibers are composed of soluble fibers (a component of the cereal cell wall), and insoluble non-cellulosic polysaccharides [60]. In this study, as expected, the drying process concentrated the MF fiber content, especially with insoluble fibers (48.08 g/100 g; Table 2). Insoluble fibers are beneficial to intestinal health and lead to an increase in fecal volume and accelerated intestinal transit, resulting in lower blood cholesterol values and the prevention of cardiovascular disease and colon cancer [61,62]. In addition to its beneficial health properties, the use of dietary fiber is important for technological processing because it reduces oxidative rancidity and improves the textural, nutritional, and sensory properties of food products [63,64].

Energy-dispersive spectroscopy (EDS) analysis revealed that MF presents eight macro- and microelements in its composition: sodium, magnesium, potassium, calcium, manganese, iron, copper, and zinc. Calcium was the prevalent mineral (76.33%), followed by magnesium (15.21%) and potassium (5.94%) (Table 2). Different results were determined by Dick et al. [19] using optical emission spectrometry to evaluate the mineral profile of *Opuntia monacantha* cactus cladode flour. The authors found potassium as the most abundant mineral, followed by calcium and magnesium. These differences can be explained by the constitution of the different species, their growth conditions, cultivars, genetic factors, harvest periods, soil compositions, and geographic differences [65], as well as the analytical method used. Even so, in accordance with the study, mandacaru cladode flour is considerably rich in these macro- and microelements compared to *Opuntia monacantha* flour.

Mineral micronutrients are essential and assist in important metabolic functions [66]. The MF matrix may be of great nutritional and functional importance for consumers. Its high content of calcium might (upon consumption) contribute to bone mineralization [2]. The high content of potassium can optimize cellular function [67], and the presence of magnesium can directly affect mitochondria and participate in the development of nerves and muscles [68]. 

**Figure 3:**
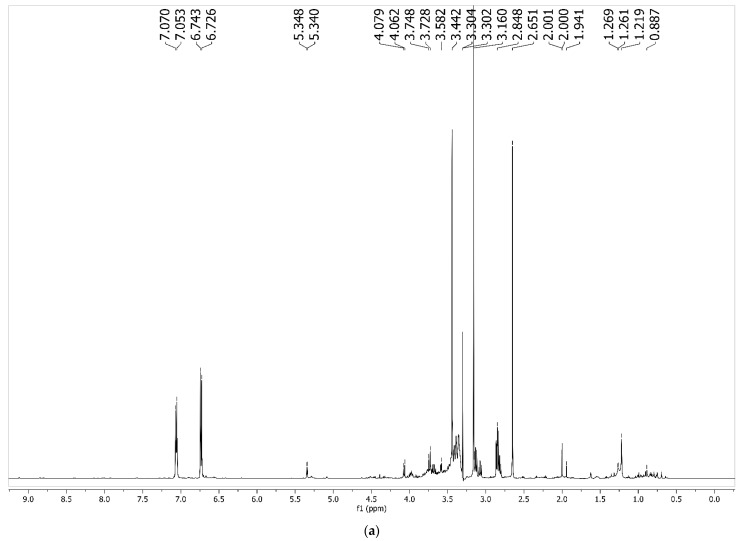

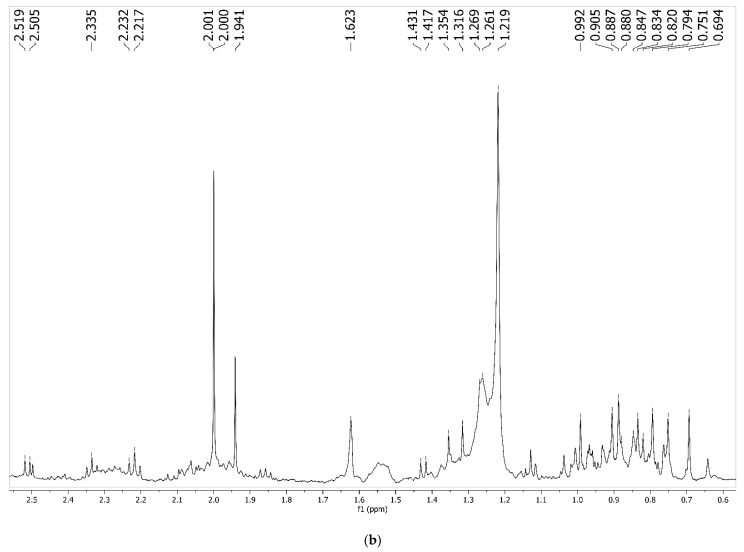

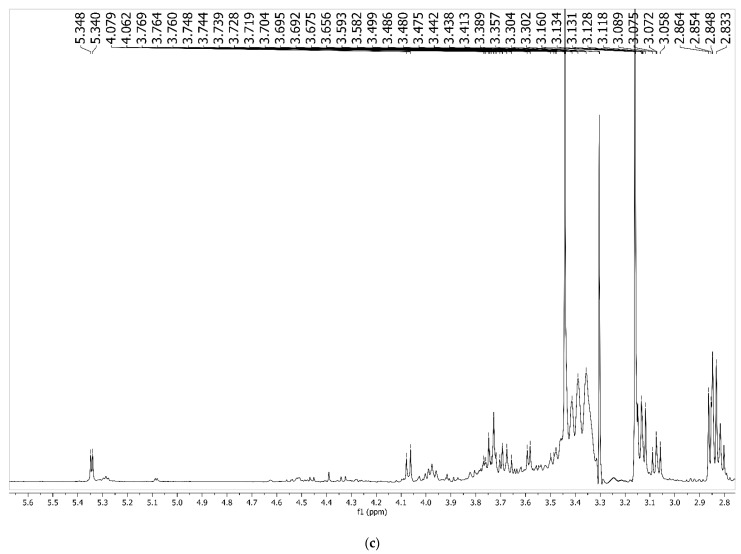

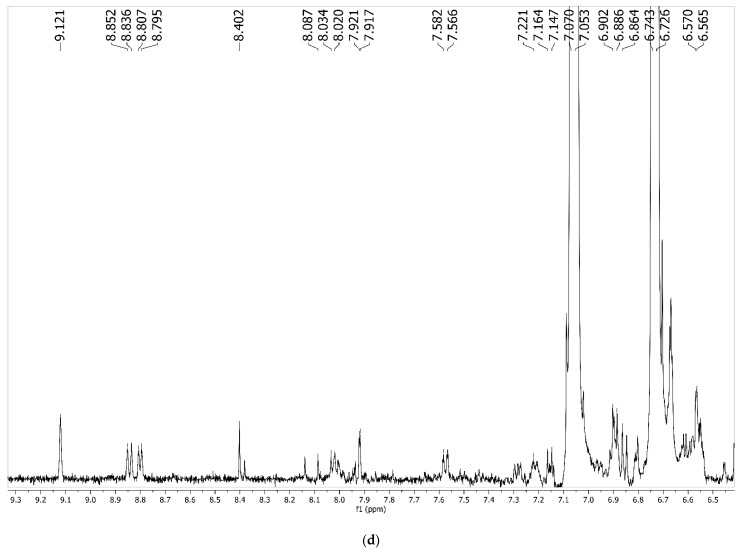
^1^H NMR spectrum of MF (**a**), ^1^H NMR spectrum expansion in the region of 2.5–0.6 ppm of MF (**b**), ^1^H NMR spectrum expansion in the region of 5.3-3.0 ppm of MF (**c**) and ^1^H NMR spectrum expansion in the region of 8.8–6.0 ppm of MF (**d**).

In the ^1^H NMR spectrum (Figure 3a), it is possible to observe the presence of signals present in the regions of δ_H_ 2.5–0.6 ppm, compatible with compounds with aliphatic carbon chains (Figure 3b). In the region of δ_H_ 5.3–3.0 ppm, the signals are compatible with hydrogens belonging to osidic units and the signals in the region of δ_H_ 8.8–6.0 ppm are compatible with aromatic compounds or with olefinic hydrogens (Figure 3c). It is still possible to identify N-methyltyramine (Figure 3d) (δ_H_ 7.08 (d, *J* = 7.8 Hz, 2H), 6.74 (d, *J* = 8.4 Hz, 2H), 2.65 (sl, 2H), 3.44 (sl, 2H), and 3.16 (s, 3H)) [69], a chemical compound already reported for this species [70]. N-methyltyramine has been popularly used in pre-workout supplements as a stimulant, similar to DMAA (dimethylamylamine), caffeine, and fat burners. In addition to being a stimulant, N-methyltyramine has been used in weight loss supplements. In this way, a fingerprint of MF was obtained via ^1^H NMR to expand the chemical characterization of its composition, as well as help in its authenticity.

### 3.3. Profile of MF Sugars and Organic Acids

In HPLC analysis (Table 3), only glucose (1.33 g/100) was detected in MF: the other sugars were below the detection limit of the method. The glucose content detected suggests the use of MF in low glycemic index diets, since mandacaru cladode flour is also rich in fiber and antioxidant compounds (Table 2 and Table 4). These bioactive compounds have been associated with the inhibition of glucose transporters in the body [71] and the restriction of carbohydrate digestion through delayed gastric emptying and enzymatic activity [72]. In general, our results corroborate the literature considering that the main sugars detected in cactus cladodes are glucose and arabinose, while those in mucilage are arabnose and xylose [73,74]. It is noteworthy that sugar composition can be influenced by the growth and development of the plant.

In the chemical, food, cosmetic, pharmaceutical, and beverage industries, organic acids (due to their various functional properties) find diversified use in biotechnological processing [75]. These acids are responsible for sensory flavor aspects and antimicrobial activity in food matrices [76,77]. In this study, citric, lactic, malic, succinic, and formic acids were detected in MF (Table 3). According to Stintzing et al. [78], the predominant organic acids in forage cactus are oxalic, malic, citric, malonic, succinic, tartaric, and piscidic acids. The principal organic acids in MF were malic (9.41 g/100 g) and citric acid (3.96 g/100 g), corroborating (in part) with a study by Chbani et al. [79] on cold-pressed *Opuntia ficus-indica* cactus that detected principally malic and quinic acids. Malic acid acts in ATP synthesis, mitochondrial respiration, and oxidative phosphorylation to enable energy release processes [80]. Citric acid acts as an important pH-adjusting acidulate, in addition to being an important antioxidant used by the food industry [81]. The obtained results reveal both the bioactive and technological potential of mandacaru flour.

### 3.4. Antioxidant Potential of MF

In view of the important biological role that antioxidant compounds play in improving human health [51,82], the discovery of alternative food sources is of great interest. The bioactivity of phenolic compounds [83], whether anti- or pro-oxidant [84], reveals the need for profiling these compounds in new food matrices, verifying their applicability in biotechnological and functional terms. Table 4 shows the MF phenolic profile, ascorbic acid content, total phenolic and flavonoid content, and antioxidant activity.

Eighteen phenolic compounds were detected in MF, with relevant amounts of the antioxidants kaempferol (99.40 mg/100 g) and myricetin (72.30 mg/100 g), which act as important antioxidants [85,86], and resveratrol (17.84 mg/100 g), which exerts anti-inflammatory activity [87]. These results suggest that MF may be used in food products to minimize the incidence of diseases related to oxidative stress (cancer, cardiovascular, and neurological diseases), while reducing the risk of chronic diseases and aging [88,89,90,91].

As a dehydrated product, we found a greater concentration of ascorbic acid (35.22 mg/100 g) in MF (Table 4), comparable to mandacaru cladode in natura (Table 1; 18.15 mg/100 g). Similar results were reported by Du Toit et al. [92], as ascorbic acid contents were lower in samples of fresh cultivars of *Opuntia ficus-indica* (14.29–28.00 mg/100 g) than in dehydrated samples (182.36–282.14 mg/100 g). Ascorbic acid, considered an important antioxidant found in high concentrations in certain plant tissues, is necessary in human food because it acts in cellular processes to prevent infections and improve immune function [93,94].

MF presented a high total phenolic content (1285.47 mg GAE/100 g) compared to the results reported in studies with *Opuntia monacantha* flour (554 mg GAE/100 g) [19] and forage palm seeds (90.2 mg/100 g) [95]. The total flavonoid content determined in MF was also significantly higher (15.19 mg CE/100 g) than that reported by Reda et al. [95] for forage cactus seeds (0.19 mg/100 g). 

The mean values of the in vitro antioxidant activity of the MF extract were higher using the FRAP method than the ABTS^•+^ method (Table 4). These antioxidant activity results may be related to the greater presence of phenolic compounds that scavenge radicals, such as myricetin, kaempferol, and resveratrol [85,86,96].

## 4. Conclusions

In this study, the mandacaru cladode dehydration process resulted in a flour that preserved its nutrients and bioactive compounds. This technological processing can expand the use of this cactus as an ingredient to develop diversified food products, adding value and increasing the knowledge of its properties. Mandacaru cladode flour presents important nutrients, with relevant mineral content (especially calcium, magnesium, and potassium) and high total fiber content (mainly insoluble fibers). In the fingerprint of the MF obtained via ^1^H NMR, it was possible to observe the presence of compounds that can differentiate this cactus species, such as N-methyltyramine. Mandacaru cladode flour also has low glucose contents and expressive amounts of malic and citric acids. The total contents of ascorbic acid, phenolic compounds, and flavonoids could be linked to its high antioxidant capacity, most likely influenced by the presence of bioactive compounds, such as myricetin, kaempferol, and resveratrol. Based on these results, mandacaru cladode is a food of great nutritional importance and a potential alternative for the expansion of agribusiness aligned with the bioeconomy. Our results reveal the potential for the beneficial use of mandacaru flour in differing food product formulations, with excellent nutritional, bioactive, and technological properties to be possibly exploited in formulations of novel functional foods. However, further study on its use as an ingredient in food products is still needed to ensure food quality and safety, especially for human consumption.

## Figures and Tables

**Table 1:** Physicochemical characteristics (media ± standard deviation) of the in natura mandacaru cladode (*Cereus jamacaru* DC.) used in the preparation of MF.

Parameters	MF
a_w_^1^	0.937 ± 0.000
Moisture (g/100 g)	13.20 ± 0.69
FMR (g/100 g) ^2^	2.66 ± 0.16
Proteins (g/100 g)	0.88 ± 0.09
Fat (g/100 g)	0.23 ± 0.09
Total carbohydrates (g/100 g)	86.24 ± 0.17
Dietary fiber (g/100 g)	
Insoluble dietary fiber	4.57 ± 0.60
Soluble dietary fiber	2.67 ± 0.61
Total dietary fiber	7.24 ± 1.20
Antioxidant potential	
Ascorbic acid (mg/100 g)	18.15 ± 0.10
Total Phenolics (mg GAE/100 g) ^3^	21.32 ± 0.52
Total Flavonoids (mg CE/100 g) ^4^	25.10 ± 0.20
FRAP (µmol Trolox TEAC/100 g) ^5^	0.89 ± 0.08
ABTS (µmol Trolox TEAC/g) ^6^	2.64 ± 0.30

^1^ a_w_—water activity; ^2^ FMR—fixed mineral residue; ^3^ results are expressed as milligrams of gallic acid equivalents (GAE) per hundred grams of sample (mg GAE/100 g); ^4^ results are expressed as milligrams of catechin equivalent (CE) per hundred grams of sample (mg CE/100 g); ^5^ results are expressed as micromol of trolox equivalent antioxidant capacity (TEAC) per hundred grams of sample (µmol TEAC/100 g). ^6^ Results are expressed as micromol of trolox equivalent antioxidant capacity (TEAC) per grams of sample (µmol TEAC/100 g). Abbreviations: FRAP—ferric-reducing ability of plasma; ABTS^•+^ cation—2,2-azino-bis (3-etilbenzo-tiazoline)-6-sulfonic acid.

**Table 2:** Physicochemical composition, dietary fiber content and mineral profile (media ± standard deviation, n: 3) of MF.

Parameters	MF
a_w_^1^	0.423 ± 0.003
Moisture (g/100 g)	8.24 ± 0.21
FMR (g/100 g) ^2^	2.82 ± 0.02
Proteins (g/100 g)	5.18 ± 0.10
Fat (g/100 g)	1.88 ± 0.14
Total Carbohydrates (g/100 g)	74.48 ± 0.20
Dietary fiber (g/100 g)	
Insoluble dietary fiber	48.08 ± 7.55
Soluble dietary fiber	0.38 ± 0.06
Total dietary fiber	48.46 ± 7.61
Macro and microelements (%)	
Na	0.95 ± 0.34
Mg	15.21 ± 0.29
K	5.94 ± 0.18
Ca	76.33 ± 0.69
Mn	0.54 ± 0.29
Fe	0.05 ± 0.29
Cu	0.59 ± 0.40
Zn	0.39 ± 0.47

a_w_^1^—water activity; FMR ^2^—fixed mineral residue.

**Table 3:** Sugar and organic acid profile (media ± standard deviation) of MF.

Parameters	MF
Simple Sugars (g/100 g)	
Glycose	1.33 ± 0.02
Fructose	<LOD
Maltose	<LOD
Rhamnose	<LOD
Organics Acids (g/100 g)	
Citric	3.96 ± 0.36
Latic	0.52 ± 0.02
Malic	9.41 ± 0.22
Succinic	1.53 ± 0.02
Formic	1.04 ± 0.07
Acetic	<LOD
Butyric	<LOD
Propionic	<LOD

<LOD: below the limit of detection.

**Table 4:** Bioactive antioxidant compound content and antioxidant activity (media ± standard deviation) of MF.

Variables	MF
Phenolic Compound Profiles (mg/100 g)	
Syringic acid	0.05 ± 0.01
Hesperidin	0.62 ± 0.05
Resveratrol	17.84 ± 1.58
Naringenin	0.21 ± 0.02
Procyanidin B1	0.79 ± 0.05
Catechin	0.45 ± 0.26
Procyanidin B2	0.13 ± 0.05
Epigallocatechin gallate	0.50 ± 0.03
Epicatequin	0.04 ± 0.05
Epicatechin gallate	2.84 ± 0.22
Procyanidin A2	0.52 ± 0.26
Chlorogenic acid	0.23 ± 0.02
Caffeic acid	0.15 ± 0.01
Trans-resveratrol	0.44 ± 0.03
Miricetin	72.30 ± 5.91
Quercitin	1.74 ± 0.34
Rutine	0.03 ± 0.02
Kaempferol	99.40 ± 8.89
Ascorbic acid (mg/100 g)	35.22 ± 0.50
Total phenolics (mg GAE/100 g) ^1^	1285.47 ± 0.10
Total flavonoids (mg CE/100 g) ^2^	15.19 ± 0.50
FRAP (µmol Trolox TEAC/100 g) ^3^	249.45 ± 0.20
ABTS (µmol Trolox TEAC/g) ^4^	0.39 ± 0.20

^1^ Results expressed in milligram gallic acid equivalents (GAE) per hundred grams of sample (mg GAE/100 g); ^2^ results expressed in milligram catechin equivalent (CE) per hundred grams of sample (mg CE/100 g); ^3^ results expressed as micromol of Trolox equivalent antioxidant capacity (TEAC) per hundred grams of sample (µmol TEAC/100 g); ^4^ results expressed as micromol of Trolox equivalent antioxidant capacity (TEAC) per grams of sample (µmol TEAC/g). Abbreviations: FRAP—ferric-reducing ability of plasma; ABTS^•+^ cation—2,2-azino-bis (3-etilbenzo-tiazoline)-6-sulfonic acid.

## Data Availability

All data generated or analyzed during this study are included in this published article.

## References

[1] Chance E., Ashton W., Pereira J., Mulrow J., Norberto J., Derrible S., Guilbert S. (2018). The Plant—An experiment in urban food sustainability. Environ. Prog. Sustain. Energy.

[2] Díaz L.D., Fernández-Ruiz V., Cámara M. (2020). An international regulatory review of food health-related claims in functional food products labeling. J. Funct. Foods.

[3] Vinusha K.S., Deepika K., Johnson T.S., Agrawal G.K., Rakwal R. (2018). Proteomic studies on lactic acid bacteria: A review. Biochem. Biophys. Rep..

[4] Conti M.V., Guzzetti L., Panzeri D., De Giuseppe R., Coccetti P., Labra M., Cena H. (2021). Bioactive compounds in legumes: Implications for sustainable nutrition and health in the elderly population. Trends Food Sci. Technol..

[5] Thamagasorn M., Pharino C. (2019). An analysis of food waste from a flight catering business for sustainable food waste management: A case study of halal food production process. J. Clean. Prod..

[6] Machado T.A.D.G., Pacheco M.T.B., Queiroga R.d.C.R.d.E., Cavalcante L.M., Bezerril F.F., Ormenese R.d.C.S.C., Garcia A.d.O., Nabeshima E.H., Pintado M.M.E., de Oliveira M.E.G. (2021). Nutritional, physicochemical and sensorial acceptance of functional cookies enriched with xiquexique (*Pilosocereus gounellei*) flour. PLoS ONE.

[7] Monteiro M.L.G., Mársico E.T., Soares Junior M.S., Magalhães A.O., Canto A.C.V.C.S., Costa-Lima B.R.C., Alvares T.S., Conte Junior C.A. (2016). Nutritional profile and chemical stability of pasta fortified with Tilapia (*Oreochromis niloticus*) flour. PLoS ONE.

[8] Araújo D.F.S., Oliveira M.E.G., Carvalho P.O.A.A., Tavares E.A., Guerra G.C.B., Queiroga R.C.R.E., Langassner S.M.Z., Bezerril F.F., Silveira A.C.S., Medeiros G.K.V.V., Jacob M.C.M., Albuquerque U.P. (2021). Food Plants in the Caatinga. Local Food Plants of Brazil, Ethnobiology.

[9] Bezerril F.F., Souza M.D.F.V.D., Lima M.D.S., Pacheco M.T.B., de Carvalho P.O.A.A., Sampaio K.B., de Sousa Y.R.F., Milani R.F., Goldbeck R., Borges G.D.S.C. (2021). Physicochemical characteristics and bioactive compounds of the Xique-xique (*Pilosocereus gounellei*) cactus from Caatinga Brazilian: Are they nutritive and functional?. J. Food Meas. Charact..

[10] Barthlott W., Hunt D.R., Kubitzki K., Rohwer J.G., Bitttich V. (1993). Cactaceae. Flowering Plants Dicotyledons: Magnoliid, Hamamelid and Caryophyllid Families.

[11] Carvalho T.K.N., De Lucena C.M., Lima J.R.F., Da Cruz D.D., De Lucena R.F.P. (2019). Local botanical knowledge of cacti in the semi-arid region of Paraíba, northeastern Brazil. Ethnobot. Res. Appl..

[12] Guerrero P.C., Majure L.C., Cornejo-Romero A., Hernández-Hernández T. (2019). Phylogenetic relationships and evolutionary trends in the cactus family. J. Hered..

[13] Schulz K., Guschal M., Kowarik I., de Almeida-Cortez J.S., Sampaio E.V.D.S.B., Cierjacks A. (2019). Grazing reduces plant species diversity of caatinga dry forests in northeastern Brazil. Appl. Veg. Sci..

[14] Soares L.M.N., Silva G.M., Buriti F.C.A., Alves H.S. (2021). *Cereus jamacaru* D.C. (Mandacaru): A promising native Brazilian fruit as a source of nutrients and bioactives derived from its pulp and skin. Plant Foods Hum. Nutr..

[15] Destaw F., Fenta M. (2021). Climate change adaptation strategies and their predictors amongst rural farmers in Ambassel district, Northern Ethiopia. J. Disaster Risk Stud..

[16] Stintzing F.C., Carle R. (2005). Cactus stems (*Opuntia* spp.): A review on their chemistry, technology, and uses. Mol. Nutr. Food Res..

[17] Bouazizi S., Montevecchi G., Antonelli A., Hamdi M. (2020). Effects of prickly pear (*Opuntia ficus-indica* L.) peel flour as an innovative ingredient in biscuits formulation. LWT Food Sci. Technol..

[18] Dick M., Limberger C., Thys R.C.S., Rios A.d.O., Flôres S.H. (2020). Mucilage and cladode flour from cactus (*Opuntia monacantha*) as alternative ingredients in gluten-free crackers. Food Chem..

[19] Chahdoura H., Chaouch M.A., Chouchéne W., Chahed A., Achour S., Adouni K., Mosbah H., Majdoub H., Flamini G., Achour L. (2018). Incorporation of *Opuntia macrorhiza* Engelm. in cake-making: Physical and sensory characteristics. LWT.

[20] Dantas D.L.d.S., Viera V.B., Soares J.K.B., dos Santos K.M.O., Egito A.S.d., Figueirêdo R.M.F., Lima M.d.S., Machado N.A.F., Souza M.d.F.V.d., da Conceição M.L. (2022). *Pilosocereus gounellei* (xique-xique) flour: Improving the nutritional, bioactive, and technological properties of probiotic goat-milk yogurt. LWT Food Sci. Technol..

[21] American Public Health Association (2015). Compendium of Methods for the Microbiological Examination of Foods.

[22] Brito T.B.N., Pereira A.P.A., Pastore G.M., Moreira R.F.A., Ferreira M.S.L., Fai A.E.C. (2020). Chemical composition and physicochemical characterization for cabbage and pineapple by-products flour valorization. LWT.

[23] AOAC (2019). Official Methods of Analysis of AOAC International.

[24] Folch J., Lees M., Sloane Stanley G.H. (1957). A simple method for the isolation and purification of total lipids from animal tissues. J. Biol. Chem..

[25] Prosky L., Asp N.-G., Schweizer T.F., Devries J.W., Furda I. (1992). Determination of insoluble and soluble dietary fiber in foods and food products. J. AOAC Int..

[26] Etienne D.T., Romuald M.M., Ysidor K.N., Daouda S., Adama C., Marius B.G.H. (2017). Nutritive contents of cakes enriched with almonds powder of Terminalia catappa of Côte d’Ivoire. Asian Res. J. Agric..

[27] Coelho E.M., Padilha C.V.d.S., Miskinis G.A., de Sá A.G.B., Pereira G.E., de Azevêdo L.C., Lima M.d.S. (2018). Simultaneous analysis of sugars and organic acids in wine and grape juices by HPLC: Method validation and characterization of products from northeast Brazil. J. Food Compos. Anal..

[28] Padilha C.V.d.S., Miskinis G.A., de Souza M.E.A.O., Pereira G.E., de Oliveira D., Bordignon-Luiz M.T., Lima M.d.S. (2017). Rapid determination of flavonoids and phenolic acids in grape juices and wines by RP-HPLC/DAD: Method validation and characterization of commercial products of the new Brazilian varieties of grape. Food Chem..

[29] Batista K.S., Alves A.F., Lima M.d.S., da Silva L.A., Lins P.P., Gomes J.A.d.S., Silva A.S., Toscano L.T., Meireles B.R.L.d.A., Cordeiro A.M.T.d.M. (2018). Beneficial effects of consumption of acerola, cashew or guava processing by-products on intestinal health and lipid metabolism in dyslipidaemic female Wistar rats. Br. J. Nutr..

[30] Lima R.d.S., Ferreira S.R.S., Vitali L., Block J.M. (2019). May the superfruit red guava and its processing waste be a potential ingredient in functional foods?. Food Res. Int..

[31] Liu M., Li X.Q., Weber C., Lee C.Y., Brown J., Liu R.H. (2002). Antioxidant and antiproliferative activities of raspberries. J. Agric. Food Chem..

[32] Zhishen J., Mengcheng T., Jianming W. (1999). The determination of flavonoid contents in mulberry and their scavenging effects on superoxide radicals. Food Chem..

[33] Benzie I.F.F., Strain J.J. (1996). The ferric reducing ability of plasma (FRAP) as a measure of “antioxidant power”: The FRAP assay. Anal. Biochem..

[34] Pulido R., Bravo L., Saura-Calixto F. (2000). Antioxidant activity of dietary polyphenols as determined by a modified ferric reducing/antioxidant power assay. J. Agric. Food Chem..

[35] Sariburun E., Şahin S., Demir C., Türkben C., Uylaşer V. (2010). Phenolic content and antioxidant activity of raspberry and blackberry cultivars. J. Food Sci..

[36] Brazil (2019). Resolução RDC nº 331, de 23 de Dezembro de 2019. Brasília: Diário Oficial da República. https://www.gov.br/agricultura/pt-br/assuntos/inspecao/produtos-vegetal/legislacao-1/biblioteca-de-normas-vinhos-e-bebidas/resolucao-rdc-no-331-de-23-de-dezembro-de-2019.pdf/view.

[37] Canalis M.S.B., León A.E., Ribotta P.D. (2019). Incorporation of dietary fiber on the cookie dough. Effects on thermal properties and water availability. Food Chem..

[38] Bressiani J., Oro T., Da Silva P., Montenegro F., Bertolin T., Gutkoski L., Gularte M. (2019). Influence of milling whole wheat grains and particle size on thermo-mechanical properties of flour using Mixolab. Czech J. Food Sci..

[39] Ma S., Wang C., Li L., Wang X. (2000). Effects of particle size on the quality attributes of wheat flour made by the milling process. Cereal Chem..

[40] Saad A.M., El-Saadony M.T., Mohamed A.S., Ahmed A.I., Sitohy M.Z. (2021). Impact of cucumber pomace fortification on the nutritional, sensorial and technological quality of soft wheat flour-based noodles. Int. J. Food Sci. Technol..

[41] Sakhare S.D., Inamdar A.A., Soumya C., Indrani D., Rao G.V. (2014). Effect of flour particle size on microstructural, rheological and physico-sensory characteristics of bread and south Indian parotta. J. Food Sci. Technol..

[42] Gómez M., Gutkoski L.C., Bravo-Núñez Á. (2020). Understanding whole-wheat flour and its effect in breads: A review. Compr. Rev. Food Sci. Food Saf..

[43] Cui R., Zhu F. (2020). Effect of ultrasound on structural and physicochemical properties of sweetpotato and wheat flours. Ultrason. SonoChem..

[44] Belorio M., Sahagún M., Gómez M. (2019). Influence of flour particle size distribution on the quality of maize gluten-free cookies. Foods.

[45] Sarker M.Z.I., Elgadir M.A., Ferdosh S., Akanda M.J.H., Aditiawati P., Noda T. (2012). Rheological behavior of starch-based biopolymer mixtures in selected processed foods. Starch-Stärke.

[46] Ray S., Raychaudhuri U., Chakraborty R. (2016). An overview of encapsulation of active compounds used in food products by drying technology. Food Biosci..

[47] Bourdoux S., Li D., Rajkovic A., Devlieghere F., Uyttendaele M. (2016). Performance of drying technologies to ensure microbial safety of dried fruits and vegetables. Compr. Rev. Food Sci. Food Saf..

[48] Selani M.M., Brazaca S.G.C., Dias C.T.S., Ratnayake W.S., Flores R.A., Bianchini A. (2014). Characterisation and potential application of pineapple pomace in an extruded product for fibre enhancement. Food Chem..

[49] Boukid F., Boukid Z., Mejri M. (2015). *Opuntia* cladodes: Physicochemical parameters, functional properties and application in formulation of rolled cake of cladode flour fabric (Part 2). Int. J. Adv. Res. Comput. Sci. Softw..

[50] Brasília: Diário Oficial da União Resolução N° 12 de Julho de 1978. https://bvsms.saude.gov.br/bvs/saudelegis/anvisa/2001/anexos/anexos_res0012_02_01_2001.pdf.

[51] Nabil B., Ouaabou R., Ouhammou M., Essaadouni L., Mahrouz M. (2020). Functional properties, antioxidant activity, and organoleptic quality of novel biscuit produced by moroccan cladode flour “*Opuntia ficus-indica*”. J. Food Qual..

[52] Xiao Y., Liu H., Wei T., Shen J., Wang M. (2017). Differences in physicochemical properties and in vitro digestibility between tartary buckwheat flour and starch modified by heat-moisture treatment. LWT Food Sci. Technol..

[53] Bilge G., Sezer B., Eseller K.E., Berberoglu H., Koksel H., Boyaci I.H. (2016). Ash analysis of flour sample by using laser-induced breakdown spectroscopy. Spectrochim. Acta Part B At. Spectrosc..

[54] Marshall M.R., Nielsen S.N. (2010). Ash Analysis. Food Analysis.

[55] Petkova N.T., Petrova I., Ivanov I., Mihov R., Hadjikinova R., Ognyanov M., Nikolova V. (2017). Nutritional and antioxidant potential of carob (*Ceratonia siliqua*) flour and evaluation of functional properties of its polysaccharide fraction. J. Pharm. Sci. Res..

[56] Argel N.S., Ranalli N., Califano A.N., Andrés S.C. (2020). Influence of partial pork meat replacement by pulse flour on physicochemical and sensory characteristics of low-fat burgers. J. Sci. Food Agric..

[57] Micale R., Giallanza A., Russo G., La Scalia G. (2017). Selection of a sustainable functional pasta enriched with *Opuntia* using ELECTRE III methodology. Sustainability.

[58] Sartori A.G.d.O., de Alencar S.M., Bastos D.H.M., Regitano d’Arce M.A.B., Skibsted L.H. (2018). Effect of water activity on lipid oxidation and nonenzymatic browning in Brazil nut flour. Eur. Food Res. Technol..

[59] Parafati L., Restuccia C., Palmeri R., Fallico B., Arena E. (2020). Characterization of prickly pear peel flour as a bioactive and functional ingredient in bread preparation. Foods.

[60] Zhang H., Wang H., Cao X., Wang J. (2018). Preparation and modification of high dietary fiber flour: A review. Food Res. Int..

[61] Deehan E.C., Duar R.M., Armet A.M., Perez-Muñoz M.E., Jin M., Walter J. (2017). Modulation of the gastrointestinal microbiome with nondigestible fermentable carbohydrates to improve human health. Microbiol. Spectr..

[62] Soliman G.A. (2019). Dietary fiber, atherosclerosis, and cardiovascular disease. Nutrients.

[63] Díaz-Vela J., Totosaus A., Pérez-Chabela M.L. (2015). Integration of agroindustrial co-products as functional food ingredients: Cactus pear (*Opuntia ficus indica*) flour and pineapple (*Ananas comosus*) peel flour as fiber source in cooked sausages inoculated with lactic acid bacteria. J. Food Process. Preserv..

[64] Sah B.N.P., Vasiljevic T., McKechnie S., Donkor O.N. (2016). Physicochemical, textural and rheological properties of probiotic yogurt fortified with fibre-rich pineapple peel powder during refrigerated storage. LWT Food Sci. Technol..

[65] Özcan M.M., Al Juhaimi F.Y. (2011). Nutritive value and chemical composition of prickly pear seeds (*Opuntia ficus indica* L.) growing in Turkey. Int. J. Food Sci. Nutr..

[66] Akram M., Munir N., Daniyal M., Egbuna C., Găman M.A., Onyekere P.F., Olatunde A., Egbuna C., Tupas G.D. (2020). Vitamins and Minerals: Types, Sources and Their Functions. Functional Foods and Nutraceuticals.

[67] Kulawiak B., Bednarczyk P., Szewczyk A. (2021). Multidimensional regulation of cardiac mitochondrial potassium channels. Cells.

[68] Liu M., Yang H., Mao Y. (2019). Magnesium and liver disease. Ann. Transl. Med..

[69] Zhao J., Wang M., Avula B., Khan I.A. (2018). Detection and quantification of phenethylamines in sports dietary supplements by NMR approach. J. Pharm. Biomed. Anal..

[70] Bruhn J.G., Lindgren J.E. (1976). Cactaceae alkaloids. XXIII. Alkaloids of *Pachycereus pecten-aboriginum* and *Cereus jamacaru*. Lloydia.

[71] Sánchez-Tapia M., Aguilar-López M., Pérez-Cruz C., Pichardo-Ontiveros E., Wang M., Donovan S.M., Tovar A.R., Torres N. (2017). Nopal (*Opuntia ficus indica*) protects from metabolic endotoxemia by modifying gut microbiota in obese rats fed high fat/sucrose diet. Scientific Reports.

[72] Qi X., Al-Ghazzewi F.H., Tester R.F. (2018). Dietary fiber, gastric emptying, and carbohydrate digestion: A mini-review. Starch-Stärke.

[73] Figueroa-Pérez M.G., Pérez-Ramírez I.F., Paredes-López O., Mondragón-Jacobo C., Reynoso-Camacho R. (2016). Phytochemical composition and in vitro analysis of nopal (*O. ficus-indica*) cladodes at different stages of maturity. Int. J. Food Prop..

[74] Medina-Torres L., Brito-de la Fuente E., Torrestiana-Sanchez B., Katthain R. (2000). Rheological properties of the mucilage gum (*Opuntia ficus indica*). Food Hydrocoll..

[75] Ata R., Aladdin A., Othman N.Z., Abd Malek R., Leng O.M., Aziz R.A., El Enshasy H. (2015). Lactic acid applications in pharmaceutical and cosmeceutical industries. J. Chem. Pharm. Res..

[76] Castro-Muñoz R. (2019). Pervaporation: The emerging technique for extracting aroma compounds from food systems. J. Food Eng..

[77] Pham V.H., Abbas W., Huang J., He Q., Zhen W., Guo Y., Wang Z. (2022). Effect of blending encapsulated essential oils and organic acids as an antibiotic growth promoter alternative on growth performance and intestinal health in broilers with necrotic enteritis. Poult. Sci..

[78] Stintzing F.C., Schieber A., Carle R. (2001). Phytochemical and nutritional significance of cactus pear. Eur. Food Res. Technol..

[79] Chbani M., Matthäus B., Charrouf Z., El Monfalouti H., Kartah B., Gharby S., Willenberg I. (2020). Characterization of phenolic compounds extracted from cold pressed cactus (*Opuntia ficus-indica* L.) seed oil and the effect of roasting on their composition. Foods.

[80] Ferreira I., Ortigoza Á., Moore P. (2019). Magnesium and malic acid supplement for fibromyalgia. Medwave.

[81] Adeyi O., Ikhu-Omoregbe D.I.O., Jideani V. (2019). Effect of citric acid on physical stability of sunflower oil-in-water emulsion stabilized by gelatinized bambara groundnut flour. Int. J. Civ. Eng..

[82] Martins N., Barros L., Ferreira I.C.F.R. (2016). In vivo antioxidant activity of phenolic compounds: Facts and gaps. Trends Food Sci. Technol..

[83] Dabulici C.M., Sârbu I., Vamanu E. (2020). The bioactive potential of functional products and bioavailability of phenolic compounds. Foods.

[84] Vieira-Frez F.C., Sehaber-Sierakowski C.C., Perles J.V.C.M., Bossolani G.D.P., Verri Jr W.A., do Nascimento R.C., Guarnier F.A., Bordini H.P., Blegniski F.P., Martins H.A. (2020). Anti-and pro-oxidant effects of quercetin stabilized by microencapsulation on interstitial cells of Cajal, nitrergic neurons and M2-like macrophages in the jejunum of diabetic rats. Neurotoxicology.

[85] Ijaz M.U., Anwar H., Iqbal S., Ismail H., Ashraf A., Mustafa S., Samad A. (2021). Protective effect of myricetin on nonylphenol-induced testicular toxicity: Biochemical, steroidogenic, hormonal, spermatogenic, and histological-based evidences. Environ. Sci. Pollut. Res..

[86] Torres-Villarreal D., Camacho A., Castro H., Ortiz-Lopez R., de la Garza A.L. (2019). Anti-obesity effects of kaempferol by inhibiting adipogenesis and increasing lipolysis in 3T3-L1 cells. J. Physiol. Biochem..

[87] Coutinho D.D.S., Pacheco M.T., Frozza R.L., Bernardi A. (2018). Anti-inflammatory effects of resveratrol: Mechanistic insights. Int. J. Mol. Sci..

[88] Santos-Díaz M.d.S., Camarena-Rangel N.G. (2019). Cacti for production of metabolites: Current state and perspectives. Appl. Microbiol. Biotechnol..

[89] Jelena C.H., Giorgio R., Justyna G., Neda M.-D., Natasa S., Artur B., Giuseppe G., Galanakis C.M. (2018). Beneficial Effects of Polyphenols on Chronic Diseases and Ageing. Polyphenols: Properties, Recovery and Applications.

[90] Jomová K., Hudecova L., Lauro P., Simunkova M., Alwasel S.H., Alhazza I.M., Valko M. (2019). A switch between antioxidant and prooxidant properties of the phenolic compounds myricetin, morin, 3′, 4′-dihydroxyflavone, taxifolin and 4-hydroxy-coumarin in the presence of copper (II) ions: A spectroscopic, absorption titration and DNA damage study. Molecules.

[91] Luna-Guevara M.L., Luna-Guevara J.J., Hernández-Carranza P., Ruíz-Espinosa H., Ochoa-Velasco C.E., Rahman A. (2018). Phenolic Compounds: A Good Choice Against Chronic Degenerative Diseases. Studies in Natural Products Chemistry.

[92] Du Toit A., De Wit M., Osthoff G., Hugo A. (2018). Antioxidant properties of fresh and processed cactus pear cladodes from selected *Opuntia ficus-indica* and *O. robusta* cultivars. South Afr. J. Bot..

[93] Bilska K., Wojciechowska N., Alipour S., Kalemba E.M. (2019). Ascorbic acid—The little-known antioxidant in woody plants. Antioxidants.

[94] Njus D., Kelley P.M., Tu Y.-J., Schlegel H.B. (2020). Ascorbic acid: The chemistry underlying its antioxidant properties. Free Radic. Biol. Med..

[95] Reda T.H., Atsbha M.K. (2019). Nutritional composition, antinutritional factors, antioxidant activities, functional properties, and sensory evaluation of cactus pear (*Opuntia ficus-indica*) seeds grown in tigray region, Ethiopia. Int. J. Food Sci..

[96] Huyut Z., Beydemir Ş., Gülçin İ. (2017). Antioxidant and antiradical properties of selected flavonoids and phenolic compounds. Biochem. Res. Int..

